# Blood‐Based Epigenetic Signatures in Brazilian Males With Alcohol Use Disorder

**DOI:** 10.1111/adb.70161

**Published:** 2026-04-28

**Authors:** Laís da Silva Pereira‐Rufino, Raissa Mazzer de Sino Romano, Regiane Chiavelli Lamim, Rafael Conte, Denise Ribeiro Gobbo, Marcelo Carvalho da Conceição, Vanessa Kiyomi Ota, Lucas Liro, Leslie Domenici Kulikowski, Adriana Marcassa Tucci, Ana Lucia de Moraes Horta, Ana Maria Sebastião, Maria Lucia Oliveira Souza‐Formigoni, João Ricardo Nickenig Vissoci, Alexandre Ferro Aissa, Isabel Cristina Céspedes

**Affiliations:** ^1^ Departamento de Morfologia e Genética, Escola Paulista de Medicina Universidade Federal de São Paulo – UNIFESP São Paulo SP Brazil; ^2^ Departamento de Oncologia Clínica e Experimental, Escola Paulista de Medicina Universidade Federal de São Paulo – UNIFESP São Paulo SP Brazil; ^3^ Departamento de Patologia, Faculdade de Medicina Universidade de São Paulo – USP São Paulo SP Brazil; ^4^ Departamento de Biociências Universidade Federal de São Paulo – UNIFESP São Paulo SP Brazil; ^5^ Departamento de Saúde Coletiva Universidade Federal de São Paulo – UNIFESP São Paulo SP Brazil; ^6^ Instituto de Farmacologia e Neurociências, Faculdade de Medicina Universidade de Lisboa Lisbon Portugal; ^7^ Centro Cardiovascular da Universidade de Lisboa, CCUL, Faculdade de Medicina Universidade de Lisboa Lisbon Portugal; ^8^ Departamento de Psicobiologia, Escola Paulista de Medicina Universidade Federal de São Paulo – UNIFESP São Paulo SP Brazil; ^9^ Duke Global Health Institute Duke University Durham North Carolina USA

**Keywords:** alcohol use disorder (AUD), central nervous system, DNA methylation, neuronal development, synaptic organization

## Abstract

Alcohol use disorder (AUD) is a chronic and progressive disease that affects individuals worldwide. AUD individuals exhibit epigenetic alterations across tissues, mainly in the brain. Despite advances in the field, there is a gap in epigenome‐wide association studies (EWAS) conducted in a Brazilian cohort that is genetically and culturally diverse. This study aimed to describe an exploratory EWAS conducted in Brazilian males with AUD and to identify DNA methylation signatures associated with brain‐related biological pathways. Peripheral blood samples were collected from 54 patients with AUD in the early stages of withdrawal (AUD group) and 70 control individuals (CON group) (Brazil, São Paulo). Genome‐wide DNA methylation was assessed using the Infinium MethylationEPIC BeadChip v2.0. We identified four differentially methylated loci in genes involved in intracellular signalling and cellular stress responses. Focusing on the nervous system, enrichment analyses revealed pathways related to neuronal/axon development and synaptic organization hypomethylated in the AUD group (*p*. adjust < 0.05). Differentially methylated regions (DMRs) showed hypomethylation in regulatory and exonic regions of *ABAT, DLX5, PHGDH, TRPM2* and *GABBR1* genes, which are within the enriched pathways. In conclusion, to the best of our knowledge, this is the first EWAS in a Brazilian cohort with AUD to identify alterations in DNA methylation, highlighting genes and pathways involved in neurogenesis, synaptic and GABAergic signalling. These epigenetic modifications suggest an impact of chronic alcohol consumption on critical biological processes of the CNS, potentially contributing to the cognitive and behavioural impairments observed in AUD. The results reinforce the importance of epigenetic studies in mixed‐race populations.

## Graphical abstract

This study represents the first epigenome‐wide association analysis of Alcohol Use Disorder (AUD) in a genetically and culturally diverse Brazilian cohort. Genome‐wide DNA methylation profiling revealed hypomethylation in genes and pathways related to neuronal development, synaptic organization, intracellular signaling and cellular stress responses in individuals with AUD. Differentially methylated regions in *ABAT*, *DLX5*, *PHGDH*, *TRPM2* and *GABBR1* highlight epigenetic disruption of neurogenesis and GABAergic signaling, suggesting a mechanistic link between chronic alcohol exposure and central nervous system dysfunction.

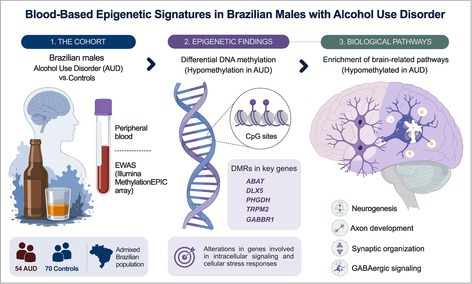

## 1 Introduction

Alcohol use disorder (AUD) is a chronic and progressive condition affecting over 400 million people worldwide. It directly impairs cognitive and emotional performance, compromising treatment success and leading to significant social and economic consequences [1]. Susceptibility to AUD is determined by genetic and environmental factors, with heritability estimated between 50% and 60% [2]. The remaining risk is determined by a complex interaction between epigenetic mechanisms and environmental factors, which play a crucial role in addiction vulnerability [3]. Epigenetic mechanisms integrate physiological and environmental stimuli to regulate chromatin structure and gene expression [4, 5, 6]. Increasing evidence indicates that chronic alcohol exposure is associated with alterations in DNA methylation [7, 8], which may influence gene expression related to cellular function and physiological processes in the brain, which leads to dysregulated synaptic plasticity [9]. As a result, individuals with AUD undergo significant alterations in neuronal function, resulting in cognitive decline and psychiatric disorders. These impairments contribute to behavioural changes observed in AUD patients and are an important factor in the reduced success rates of treatments [10]. Although the influence of alcohol on the epigenome is recognized, the current literature focuses largely on European populations [4, 11, 12, 13]. There is a significant gap in the study of AUD in Latino samples, especially Brazilians, whose population is highly mixed, with European, African, Asian and Native American influences [14, 15]. Beyond genetic admixture, cultural factors are also relevant, as the Brazilian population exhibits distinct drinking patterns shaped by social, historical and cultural diversity [16]. Such differences may influence not only the prevalence of alcohol use but also its clinical manifestations and consequences. So, the lack of representation limits the understanding of the epigenetic mechanisms associated with AUD in genetically and culturally diverse populations. Therefore, to our knowledge, this is the first exploratory epigenome‐wide association study (EWAS) conducted in Brazilian males with a clinical diagnosis of AUD. By examining DNA methylation patterns in blood samples, we identified epigenetic signatures associated with brain‐related biological pathways. We also contributed to increasing diversity in epigenetic studies, highlighting the importance of including underrepresented populations in scientific research.

## 2 Methods

### 2.1 Participants

Patients with AUD (AUD group; *n* = 54) in the early stages of withdrawal (8–14 days of abstinence) and low‐risk alcohol use individuals—controls (CON group; *n* = 70) were recruited for the study (Table 1). The AUD group was in treatment at the Centro de Tratamento Bezerra de Menezes (São Paulo, SP, Brazil) and had a clinical diagnosis of AUD from the hospital staff and scored ≥ 27 on the Alcohol Smoking and Substance Involvement Screening Test (ASSIST). The CON group was recruited from Hospital São Paulo (Universidade Federal de São Paulo—UNIFESP; São Paulo, SP, Brazil) and reported none/occasional substance use (score ≤ 3) according to the ASSIST (Table 1). Sociodemographic and clinical data (previous treatment and family history of AUD) were acquired during the screening interview. Male individuals aged between 21 and 55 years were included. Individuals excluded the following: diagnosis of schizophrenia, trauma or cranial surgery, neurological disease, stroke or major physical health conditions (e.g., heart, liver or renal diseases). All individuals signed the Informed Consent Form approved by the Research Ethics Committee of UNIFESP (no. 0473/2018).

**TABLE 1:** Substance use, demographic and clinical characteristics of participants of the alcohol use disorders (AUD) and control (CON) groups.

	Groups			
Variables (median (IQR) or %)	Total *N* = 124	AUD *n* = 54	CON *n* = 70	*p* ^a^
ASSIST scores				
Tobacco	—	12.0 (22)	0.0 (0.0)	**< 0.001**
Alcohol	—	31 (7.0)	2.0 (1.0)	**< 0.001**
Cocaine	—	0.0 (2.0)	0.0 (0.0)	**< 0.001**
Age in years	38.0 (11)	40.0 (5.0)	34.0 (12.0)	**< 0.001**
Education level in years	13.0 (4.0)	11.0 (4.0)	15.0 (2.0)	**< 0.001**
Ethnicity				0.300
White	70 (56.4%)	28 (51.9%)	42 (60%)	
Mixed race^b^	27 (21.8%)	14 (26%)	13 (18.6%)	
Black	17 (13.8%)	8 (14.8%)	9 (12.8%)	
Asian	5 (4.0%)	1 (1.8%)	4 (5.7%)	
Missing data	5 (4.0%)	3 (5.5%)	2 (2.9%)	
Marital status				**0.021**
Single	51 (41%)	16 (29.7%)	35 (50%)	
Married	58 (47%)	27 (50%)	31 (44%)	
Separated	14 (11.2%)	10 (18.5%)	4 (5.7%)	
Missing data	1 (0.8%)	1 (1.8%)	0 (0%)	
Previous treatments for SUD	24 (20%)	24 (46%)	0 (0%)	**< 0.001**
Family history of SUD	54 (44%)	33 (62%)	21 (30%)	**< 0.001**
Missing data	1 (0.8%)	1	0	

*Note:* Values in bold highlight the significant data.

Abbreviations: AUD, alcohol use disorder group; CON, control group; SUD, substance use disorder.

^a^ Mann–Whitney test or Fisher's exact test or Pearson's Chi‐squared test.

^b^ The Brazilian census uses the term ‘pardos’ to refer to individuals of mixed racial background, primarily of European, African and Indigenous ancestry.

### 2.2 Substance Use Disorders Classification

ASSIST is used to assess psychoactive substance use. Scores categorize risk as low (0–3 for other substances; 0–10 for alcohol), moderate [11, 12, 13, 14, 15, 16, 17, 18, 19, 20, 21, 22, 23, 24, 25, 26] or high (≥ 27), with a maximum score of 39. In this study, scores for cannabis, cocaine and tobacco reflect the sum for each substance category [17].

### 2.3 Blood Samples

Blood samples were collected in EDTA tubes and stored at −20°C until DNA extraction (PureGene kit, QIAGEN, USA). DNA concentration (> 300 ng/μL) was quantified using the Qubit dsDNA HS Assay Kit (Thermo Fisher Scientific, USA). DNA purity was assessed using a NanoDrop spectrophotometer (Thermo Fisher Scientific, USA) by the A260/280 absorbance ratio, and only samples with values between 1.8 and 2.0 were included. DNA samples were diluted to 20 ng/μL for methylation analysis.

### 2.4 Methylation EPIC Array Processing and Differential Methylation Analysis

Bisulfite conversion was performed using the Puregene Blood Kit (QIAGEN). Converted DNA was hybridized overnight to the Infinium MethylationEPIC BeadChip v2.0 (EPICv2), covering 937 055 CpG sites (Illumina, USA). BeadChips were scanned using the iScan system (Illumina, USA). Raw .*idat* files from the iScan system were processed for quality control (QC) using the Meffil R package (v1.3.7) with default settings [18].

QC metrics included detection *p*‐values, bead counts, signal‐intensity outliers, sex concordance, control probe deviations and SNP concordance. All QC thresholds applied were as follows: probes with detection *p*‐values > 0.01, probes with fewer than three beads and samples showing discrepancies between reported and inferred sex, methylated or unmethylated signal outliers exceeding three standard deviations or control probe deviations exceeding five standard deviations. Samples failing any QC criterion were excluded from downstream analyses. SNP concordance was also assessed, and samples with concordance below 0.9 were flagged for removal. After removing low‐quality samples and probes, quantile normalization was performed using the first 10 technical principal components (PCs), generating beta values and *M*‐values for downstream analyses. Probes known to be cross‐reactive, containing SNPs, or outside the EPICv2 target regions, based on the hg38 reference genome, were excluded to ensure high‐quality and reliable DNA methylation measurements [19]. Finally, blood cell‐type composition was estimated from the normalized data using the reference ‘blood gse35069’ and incorporated into the final metadata for downstream adjustment [20]. All analyses were conducted in R (v4.3.2).

### 2.5 Statistical Analysis

#### 2.5.1 Differential Methylation Analyses

After preprocessing, beta values were converted to *M*‐values to improve statistical robustness in differential methylation analyses [21]. CpG sites containing missing values were evaluated, and probes with more than 5% missing data across samples were excluded prior to downstream analysis. Differential methylation analysis was conducted using the limma framework, fitting linear models to M‐values [22]. Covariates were selected a priori based on biological relevance and study design, including age, tobacco and marijuana exposure scores, technical batch and estimated blood cell‐type proportions (CD8+ T cells, CD4+ T cells and granulocytes) [23, 24]. To guide the inclusion of cellular covariates and reduce redundancy, a principal component analysis (PCA) was performed on the estimated blood cell‐type proportions (CD8+ T cells, CD4+ T cells, granulocytes, monocytes and B cells). Cell types contributing most strongly to overall cellular heterogeneity, as indicated by loadings from the first PCs, were subsequently included in the regression models [24]. Model stability was assessed by evaluating multicollinearity using variance inflation factors (VIFs) and overall test‐statistic inflation using the genomic inflation factor (*λ* = 1.03), ensuring appropriate control of confounding and robust statistical inference (Figure S1). Multiple testing correction was applied using the Benjamini–Hochberg false discovery rate (FDR) procedure, and CpG sites with FDR‐adjusted *p*‐values < 0.05 were considered statistically significant. Interpretation and visualization focused on FDR‐significant CpGs.

For biological interpretation, CpG probes were annotated and mapped to genomic coordinates using the Illumina EPIC v2 manifest file (IlluminaHumanMethylationEPICv2anno.20a1.hg38). In addition, we performed sensitivity analyses that included self‐reported ethnicity as a covariate in the regression models (Table S1).

Differentially methylated regions (DMRs) were identified using two complementary region‐based approaches. Primary regional analysis was performed using the DMRcate package [25], which identifies DMRs by applying kernel smoothing to spatially correlated CpG‐level statistics derived from linear models. In parallel, an exploratory regional analysis was conducted using the function from the sesame package. In both approaches, regional significance was evaluated using multiple testing correction at the regional level. Only DMRs with an adjusted *p*‐value < 0.05 were considered statistically significant. For the sesame‐based DMR analysis, regions were further filtered to retain those comprising at least two CpG sites: a maximum inter‐CpG distance of ≤ 500 bp and a consistent direction of methylation change across the region (≥ 80%). Finally, multiple significant segments sharing the same genomic start position were collapsed and treated as a single DMR, as they represent the same underlying methylation region.

To investigate biological functions, gene set enrichment analysis (GSEA) was performed using genes and corresponding effect sizes [26]. All CpGs with nominal *p* < 0.05 (unadjusted) were used to generate a ranked list, as recommended to preserve biological signal prior to multiple testing correction [27, 28, 29]. This approach avoids overpenalization and loss of biological signal, which can occur when strict multiple testing correction is applied prior to GSEA. GSEA was conducted with the ClusterProfiler package (v4.12.16) [30], using curated gene sets from MSigDB (v2023.2.Hs) [26, 31]. Only gene sets with FDR‐adjusted *p*‐values (*p*. adjust < 0.05) were deemed significant. This strategy is widely applied in EWAS and other high‐dimensional epigenomic studies to capture coordinated pathway‐level effects that may not reach genome‐wide significance at individual CpG sites.

#### 2.5.2 Clinical and Demographic Variables

Differences in continuous variables were assessed using the Mann–Whitney *U* test (results reported as median and interquartile range [IQR] due to nonnormal distribution). Categorical variables were compared using the Chi‐square test or Fisher's exact test (counts and percentages reported).

## 3 Results

### 3.1 Substance Use, Demographic and Clinical Characteristics

All individuals in the CON group (*n* = 70) were classified as low‐risk alcohol users (ASSIST ≤ 10), while all AUD individuals (*n* = 54) scored ≥ 27, indicating high‐risk use. Within the CON group, 45 individuals reported tobacco use, and 7 individuals reported marijuana use. None reported cocaine use. In the AUD group, 33 individuals reported tobacco use, 13 reported marijuana use and 16 reported cocaine use. In both groups, the median for these substances indicated low‐risk use. In the AUD group, 46% had prior treatment for AUD, and 62% reported a family history of AUD (compared to 0% and 30% in the CON group, respectively). The AUD group was 40.0 ± 5.0 years old, 51.9% self‐identified as white, 50% were married and had an average of 11.0 ± 4.0 years of education (Table 1).

### 3.2 Overview of DMLs Across the Genome

**FIGURE 1:**
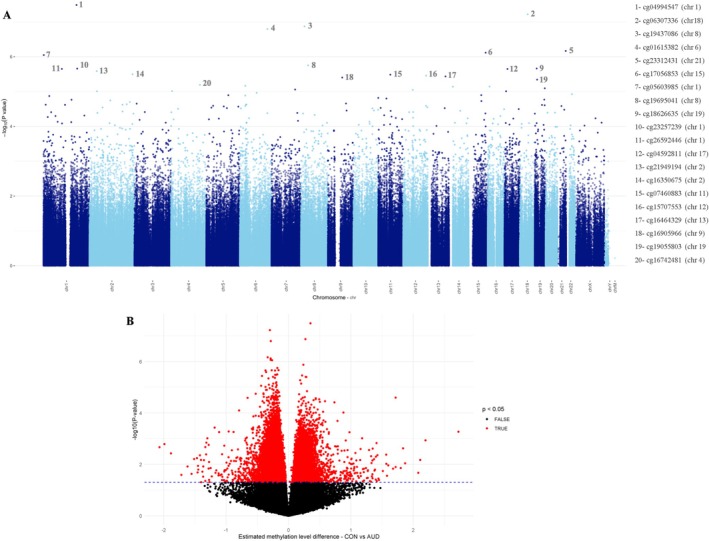
Differentially methylated loci (DMLs) associated with Alcohol Use Disorder (AUD). (A) Manhattan plot displaying the statistical significance of methylation changes across all autosomes. Each dot represents a DMLs tested in the EPICv2 array; the *X*‐axis indicates chromosomal position, and the *Y*‐axis shows the –log₁₀ (*p*‐value). (B) Volcano plot illustrating differentially methylated loci (DMLs) based on the estimated methylation level difference between the AUD and control (CON) groups (*X*‐axis) and statistical significance (−log₁₀ *p*‐value, *Y*‐axis). Red dots indicate loci with *p*‐value < 0.05 in the AUD group. Chr, chromosome.

We identified four differentially methylated loci (DMLs) (*p*. adjust < 0.05) in the AUD group compared to the CON group. The Manhattan plot (Figure 1A) displays the *p*‐values (−log10) for methylation at various CpG loci across the chromosomes. Volcano plot shows the methylation differences and the *p*‐values of the most significant DMLs (Figure 1B). Among the CpG loci identified, those associated with genes such as *UST* (Est = −0.28, adj.*p*‐value = 0.037; Δβ = −0.053) and *PIK3C3* (Est = −0.29, *p*‐adj. *p*‐value = 0.028; Δβ = −0.013) stood out as hypomethylated; while the *CSGALNACT1 (*Est = 0.27, adj.*p*‐value = 0.037; Δβ = 0.012), was hypermethylated. All the DMLs are listed in Table S2.

The sensibility analyses showed that while the inclusion of ethnicity attenuated statistical significance at the genome‐wide level, this attenuation was not driven by a direct confounding effect of ethnicity. No CpG site showed a significant main effect of ethnicity, and ethnicity explained substantially less variance than case–control status. Moreover, all top CpG sites exhibited consistent directional effects across ethnic groups, and random‐effects meta‐analysis across groups supported the overall robustness of the associations, despite reduced power in minority subgroups. These findings suggest that the observed attenuation reflects quantitative heterogeneity and reduced statistical power rather than classical confounding by ethnicity.

Moreover, we identified one DMR associated with the experimental group (HMFDR < 0.05) in DMRcate analysis, overlapping the *ABAT* gene, which was hypomethylated in individuals with AUD compared to controls. In the exploratory sesame‐based DMR analysis, we identified 30 DMRs associated with the experimental group, of which 25 were hypomethylated and 5 were hypomethylated in individuals with AUD compared with controls. We found the same DMR overlapping the *ABAT* gene as the DMRcate analyses (Table S3).

### 3.3 DMLs Associated With Genetic Pathways Related to Alcohol Consumption

**FIGURE 2:**
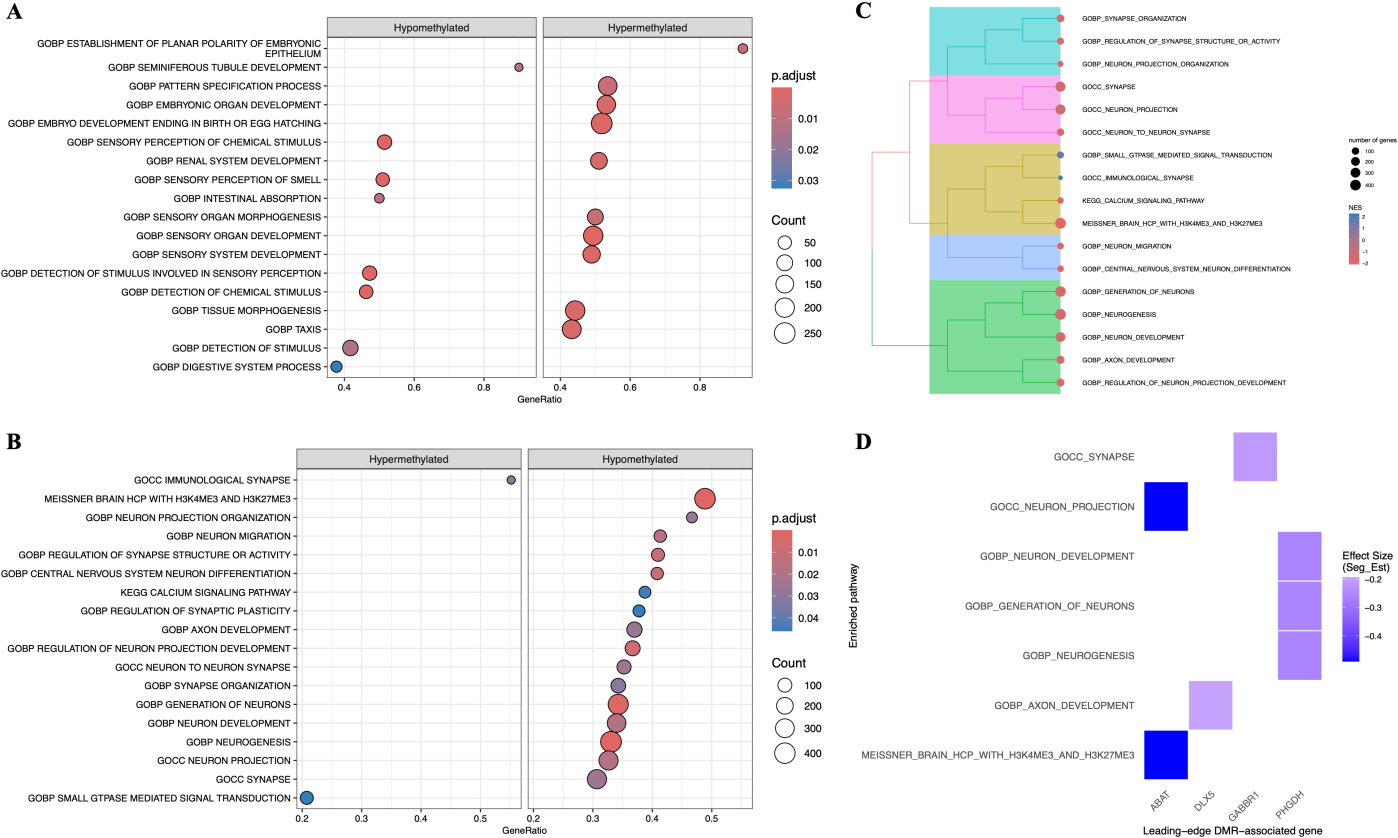
Differentially methylated loci (DMLs) in AUD group were enriched in biological pathways related to neurogenesis, synaptic organization and neuronal signalling.(A) Dot plot showing enriched standardized functional categories derived from the Gene Ontology Consortium terms (Biological Process [GOBP], Cellular Component [GOCC], Molecular Function [GOMF]) and Human Phenotype Ontology (HP) terms associated with differentially methylated loci (DMLs) in the AUD group. (B) Dot plot showing enriched gene sets based on Gene Ontology Consortium—GOBP, GOCC, Reactome, and curated datasets (e.g., Descartes, FAN, Meissner and Manno), using DMLs between alcohol use disorder (AUD) and healthy control (CON) groups. Pathways are grouped as hypomethylated or hypermethylated in the AUD group. The dot size represents the gene count, and the colour denotes the adjusted *p*‐values (FDR). (C) Treeplot depicting the hierarchical clustering of the enriched pathways from panel B, based on semantic similarity, revealing functional convergence among neurodevelopmental and synaptic terms. (D) Overlap between the most significant genes from nervous system–related enriched pathways and genes associated with differentially methylated regions (DMRs) identified using the exploratory sesame‐based analysis. Each tile represents a gene–pathway association, coloured by the segmented effect size (Seg_Est).

We conducted a GSEA with the genes associated with DMLs to identify genetic pathways related to alcohol consumption. Among hypomethylated regions (*p*. adjust < 0.05), enriched pathways were primarily related to sensory perception, chemical stimulus detection and tissue morphogenesis. In hypermethylated regions, significant pathways were predominantly associated with embryonic organ development, pattern specification, and organogenesis (Figure 2A).

### 3.4 DMLs Associated With Genetic Pathways Related to the Nervous System

We performed a focused GSEA to explore biological processes specifically related to the nervous system. This analysis included ontological categories, canonical signalling pathways and chemical genetic perturbation datasets (Figure 2B). We selected statistically significant terms associated with brain processes, neural structures and synaptic functions, aiming to establish a connection between alcohol consumption and DNA methylation that affects the nervous system.

The results revealed significant enrichment in terms related to neuronal development, including Gene Ontology Biological Process (GOBP; Gene Ontology Consortium) terms such as Generation of Neurons, Neurogenesis, Neuron Development, Neuron Migration, Neuron Projection Organization and Axon Development. Enrichment was also observed for synaptic and axonal organization, exemplified by Gene Ontology Cellular Component (GOCC) terms such as Synapse, Synapse Organization, and Neuron Projection (Figure 2B). Additionally, terms associated with bivalently promoters in brain tissue, such as Meissner Brain Hcp with H3K4ME3 and H3K27ME, were prominent. All these terms showed a hypomethylated profile in the AUD group compared to the CON group. On the other hand, hypermethylated terms were found in specific enrichment terms related to synapse (GOCC Immunological synapse) and cellular process controlled by GTPases (GOBP Small GTPase Mediated Signal Transduction) (Figure 2B).

To better understand functional convergence among these enriched pathways, Figure 2C presents a Treeplot of enriched biological pathways (GO or Reactome, etc.) clustered by functional similarity, illustrating how the enriched terms are related to one another.

### 3.5 DMRs Show Enrichment in Biological Pathways Related to Neurogenesis, Synaptic Organization and Axonal Development

Based on the enrichment analysis of biological pathways associated with brain‐related functions, we selected representative genes from each pathway for detailed description. These genes were identified from sesame‐based DMR analysis (adjusted *p*‐value < 0.05) that overlapped with the significant gene ontology (GO) terms. In the pathways related to neuronal development and axonal growth (GOBP Axon Development, GOBP Generation of Neurons, GOBP Neurogenesis, GOBP Neuron Development), DMRs mapped to the genes *DLX5* and *PHGDH*. Genes associated with neuronal projection processes (GOCC Neuron Projection, GOCC Synapse) included DMRs mapped to *ABAT*, *TRPM2* and *GABBR1*. Additionally, pathways related to H3K4me3 and H3K27me3 in brain tissue (MEISSNER Brain HCP with H3K4me3 and H3K27me3) show DMRs mapped to *ABAT* and *DLX5* (Figure 2D **).** These regions showed hypomethylation in regulatory (5′UTR) and exonic regions in the AUD group compared to controls (Table 2).

**TABLE 2:** —Genes related to DMR in biological pathways of neural development, axonal development and synaptic organization hypomethylated in the AUD group compared to the CON group.

Description	Gene	Seg ID	Estimate	*p*‐value adjusted	Position	Function
GOBP axon development	*DLX5*	148 270	−0.19	3.22e‐02	TSS1500	Neuron organization
GOBP generation of neurons	*PHGDH*	20 209	−0.26	1.85e‐03	Exon/TSS1500	Neuron diferenctiation and proliferation
	*DLX5*	148 270	−0.19	1.80e‐3	TSS1500	Neuronal differentiation
GOBP neurogenesis	*PHGDH*	394 068	−0.26	2.47e‐04	Exon/TSS1500	Neuronal maturation
	*DLX5*	40 471	−0.19	2.51e‐04	TSS1500	Neuron organization
GOBP neuron development	*PHGDH*	394 068	−0.26	8.27e‐03	5´UTR/Ex/TSS200	Plasticity
	*DLX5*	40 471	−0.19	8.35e‐03	TSS1500	Neuronal maturation
GOCC neuron projection	*ABAT*	276 565	−0.48	2.26e‐02	TSS1500/TSS200	Neuronal projections and excitability
GOCC synapse	*GABBR1*	121 148	−0.0.21	2.23e‐02	Exon/3UTR/TSS200	Inhibitory synaptic signalling

*Note:* Gene ontology categories are based on curated gene sets from the Molecular Signatures Database (MSigDB, Broad Institute) [30].

Abbreviations: 5′UTR: 5′untranslated region; AUD, alcohol use disorder; CON, control group; Ex, exon overlap; GOBP, Gene Ontology Biological (standardized functional categories derived from the Gene Ontology Consortium); TSS200 = 0–200 bases upstream of the transcription start site (TSS); TSS1500 = 200–1500 bases upstream of the TSS.

## 4 Discussion

To the best of our knowledge, this is the first EWAS conducted in Brazilian males with AUD identifying blood‐based DNA methylation alterations associated with chronic alcohol consumption and enriched in brain‐related biological pathways. We identified four differentially methylated CpG loci, highlighting genes involved in intracellular signalling and cellular stress responses. Focusing on the nervous system, enrichment analyses revealed pathways related to neuronal/axon development and synaptic organization hypomethylated in the AUD group. DMRs analysis showed hypomethylation in regulatory and exonic regions of *ABAT, DLX5, PHGDH, TRPM2* and *GABBR1* genes, which are within the enriched pathways.

The four DMLs identified map to genes, including *UST*, *PIK3C3* and *CSGALNACT1*. To our knowledge, none have been previously reported in the EWAS literature with AUD patients [4, 32, 33, 34]. This limited overlap may be explained not only by sample size differences but also by ancestry, environmental exposures and sociocultural factors, reinforcing the importance of conducting epigenetic studies in admixed populations [15, 16].

Furthermore, our analysis extends to pathway‐level interpretation, revealing a modulation in neurodevelopmental and synaptic processes. Specific biological pathways related to alcohol consumption and the nervous system showed a hypomethylated profile in the AUD group, including those related to neuronal development, neurogenesis, neuronal migration, axon development and synaptic organization. These findings are consistent with previous EWAS studies showing that alcohol exposure affects genes involved in neurodevelopmental and synaptic processes [11, 35, 36, 37].

Beyond single CpG associations, regional analyses identified a robust DMR overlapping the *ABAT* gene, detected by the DMRcate approach and characterized by hypomethylation in individuals with AUD. *ABAT* encodes 4‐aminobutyrate aminotransferase, a mitochondrial enzyme responsible for GABA catabolism, playing a key role in inhibitory neurotransmission [38]. Hypomethylation of *ABAT* is biologically consistent with evidence implicating γ‐aminobutyric acid (GABAergic) dysregulation as a core mechanism underlying alcohol dependence, tolerance and withdrawal [39, 40]. Chronic alcohol exposure enhances GABAergic signalling, while prolonged abstinence induces compensatory adaptations that contribute to hyperexcitability and relapse vulnerability [41]. Epigenetic modulation of *ABAT* may represent a potential molecular link between chronic alcohol exposure and long‐lasting alterations in inhibitory neurotransmission.

In addition, we performed an exploratory sesame‐based DMR analysis that identified 30 DMRs associated with the AUD group. This exploratory analysis independently replicated the DMR overlapping the *ABAT* gene, as well as the *DLX5, PHGDH, TRPM2* and *GABBR1* genes, which were integrated into enriched brain‐related pathways. *DLX5* is a transcription factor essential for GABAergic interneuron development and cortical patterning. Dysregulation of *DLX5* genes has been associated with the intensity of anxiety and compulsivity‐like behaviours [42, 43]. *PHGDH*, which encodes the rate‐limiting enzyme in serine biosynthesis, is critical for neuronal proliferation, synaptic function and redox balance. Altered regulation of serine metabolism has been linked with glial metabolism, synaptic activity and plasticity, and neurodegenerative diseases, common features in chronic AUD [44, 45]. *TRPM2* is a calcium‐permeable channel activated by oxidative stress and has been linked to alcohol‐induced neurotoxicity and neuroinflammation [46]. Meanwhile, *GABBR1* encodes the GABA‐B receptor subunit 1, directly linking our epigenetic findings to inhibitory neurotransmission and alcohol's primary pharmacological targets [47].

An important consideration in interpreting our findings is the role of abstinence. Participants were undergoing treatment and had experienced a short period of abstinence (8–14 days of abstinence), which may influence DNA methylation patterns. The literature on epigenetic changes during alcohol abstinence remains inconsistent. Some longitudinal studies describe significant differences in methylation profiles after weeks to months of abstinence [48, 49], whereas others demonstrate persistent differences during withdrawal [50, 51]. These discrepancies likely reflect differences in study design, tissue type, duration of abstinence and analytical strategies.

Despite revealing associations between AUD and DNA methylation, this study has some limitations: (1) the sample size, while adequate for exploratory analyses, limits statistical power to detect small effects; (2) the exclusive inclusion of males restricts generalizability to females, a choice made to minimize biological variability and control for hormonal confounding. Future studies should include women and compare across gender using appropriate stratification; (3) although a large proportion of participants self‐identified as white, self‐reported race in Brazil does not directly correspond to genetic ancestry, as the population is highly admixed. The lack of genome‐wide genetic data precluded direct estimation of individual ancestry proportions and explicit modelling of ancestry‐related effects. Future studies integrating genetic ancestry markers are needed to further address ancestry‐related variability; (5) while differential methylation may influence gene expression, further postmortem brain tissue functional studies are necessary to confirm biological effects in AUD; (6) as the study is exploratory, no a priori power calculation was performed. The sample size was determined by the available data, and the results should be interpreted accordingly, acknowledging that larger studies are required to confirm these findings; (7) DNA methylation is tissue‐specific, so blood‐based patterns may not fully reflect brain epigenetic changes. Thus, the findings presented should be interpreted with caution, and further investigations are needed to validate the associations identified.

In conclusion, to the best of our knowledge, this is the first EWAS in a Brazilian cohort with AUD to identify alterations in DNA methylation, highlighting genes and pathways involved in neurogenesis, synaptic and GABAergic signalling. These epigenetic modifications suggest an impact of chronic alcohol consumption on critical biological processes of the CNS, potentially contributing to the cognitive and behavioural impairments observed in AUD. The results reinforce the importance of epigenetic studies in mixed‐race populations.

## Author Contributions

**Laís da Silva Pereira‐Rufino:** conceptualization, data acquisition, data processing and analysis, formal analysis, writing – original draft. **Raissa Mazzer de Sino Romano:** data acquisition, data processing. **Regiane Chiavelli Lamim:** data acquisition, visualization. **Rafael Conte:** conceptualization, data acquisition. **Denise Ribeiro Gobbo:** conceptualization, data acquisition. **Marcelo Carvalho da Conceição:** data acquisition. **Vanessa Kiyomi Ota:** methodology, formal analysis. **Lucas Liro:** data processing. **Leslie Domenici Kulikowsk:** data processing. **Adriana Marcassa Tucci:** funding acquisition. **Ana Lucia de Moraes Horta:** funding acquisition. **Ana Maria Sebastião:** conceptualization, writing – original draft. **Maria Lucia Oliveira Souza‐Formigoni:** resources, funding acquisition, conceptualization, writing – original draft. **João Ricardo Nickenig Vissoci:** methodology, formal analysis. **Alexandre Ferro Aissa:** methodology, formal analysis. **Isabel Cristina Céspedes:** conceptualization, project administration, funding acquisition, formal analysis, data curation, writing – original draft.

## Funding

This study was financed by the São Paulo Research Foundation (FAPESP), Brazil, processes numbers 2013/01158‐7, 2014/27198‐8, 2021/13092‐7 and 2023/13554‐6 and CAPES PrInt UNIFESP processes 88887.310780/2018‐00, 88887.310785/2018‐00, 88881.310787/2018‐01 and 88887.695808/2022‐00.

## Conflicts of Interest

The authors declare no conflicts of interest.

## Supporting information


**Figure S1:** Assessment of genomic inflation A). The graph illustrates the expected versus observed distribution (−log10 *p*‐values) for the association between methylation and alcohol exposure across the epigenome. The genomic inflation factor (*λ* = 1.03) indicates moderate inflation.

**Table S1:** Sensitivity analysis assessing the impact of ethnicity on differentially methylated CpG sites Δβ, effect sizes; *p‐value from random‐effects meta‐analysis combining effects across all ethnic groups; Δ*R* ^2^, proportion of variance explained by ethnicity.

**Table S2:** CpG sites identified in the EWAS of alcohol use disorder. Chr, chromosome; CpG, cytosine‐phosphate‐guanine CpG islands are regions of approximately 1000 base pairs long, enriched in CpG dinucleotides. In humans, 72% of gene promoters contain CpG islands. CpG can then be localized toward these islands: Island, Shelf, Shore and OpenSea. Gene region is divided into several regions: TSS200 = 0–200 bases upstream of the transcription start site (TSS); TSS1500 = 200–1500 bases upstream of the TSS; 5’UTR = in the 5′ untranslated region, between the TS and the ATG start site; Body = between the ATG and the stop codon, regardless of the presence of introns, exons, TSS or promoters; 3′UTR = between the stop codon and the poly A signal.

**Table S3:** DMRs identified in the EWAS of alcohol use disorder. Chr, chromosome.

## Data Availability

Research data are not shared.
