# Supplementary figures and images for: Blood‐Based Epigenetic Signatures in Brazilian Males With Alcohol Use Disorder
Source: Addict Biol. 2026 Apr 28;31(5):e70161. doi: 10.1111/adb.70161 (PMC13122561; doi:10.1111/adb.70161)

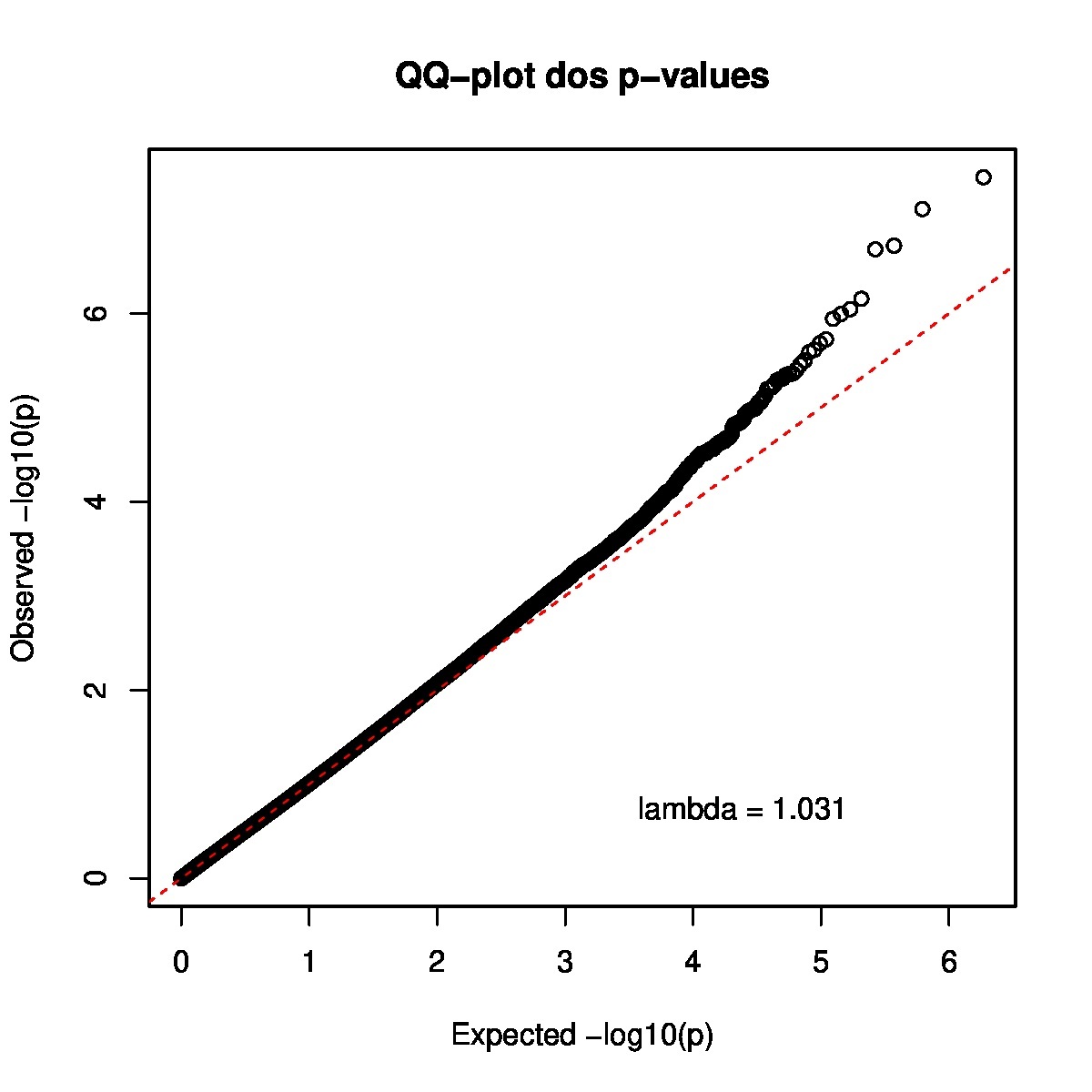

Supplement: Supplementary file 1 — Figure S1: Assessment of genomic inflation A). The graph illustrates the expected versus observed distribution (−log10 p‐values) for the association between methylation and alcohol exposure across the epigenome. The genomic inflation factor (λ = 1.03) indicates moderate inflation. [file ADB-31-e70161-s003.jpg]
